# Association of Pneumococcal and Influenza Vaccination With Patient–Physician Communication in Older Adults: A Nationwide Cross-sectional Study From the JAGES 2016

**DOI:** 10.2188/jea.JE20200505

**Published:** 2022-09-05

**Authors:** Koryu Sato, Naoki Kondo, Chiyoe Murata, Yugo Shobugawa, Kousuke Saito, Katsunori Kondo

**Affiliations:** 1 Department of Social Epidemiology, Graduate School of Medicine and School of Public Health, Kyoto University, Kyoto, Japan; 2 Department of Health Education and Health Sociology, School of Public Health, The University of Tokyo, Tokyo, Japan; 3 Department of Nutrition, School of Health and Nutrition, Tokai Gakuen University, Aichi, Japan; 4 Department of Active Aging, Graduate School of Medical and Dental Sciences, Niigata University, Niigata, Japan; 5 Department of Social Preventive Medical Sciences, Center for Preventive Medical Sciences, Chiba University, Chiba, Japan; 6 Department of Gerontological Evaluation, Center for Gerontology and Social Science, National Center for Geriatrics and Gerontology, Aichi, Japan

**Keywords:** patient–physician communication, shared decision-making, pneumococcal vaccine, influenza vaccine, older adults

## Abstract

**Background:**

Increasing the coverage of vaccinations recommended by the World Health Organization in the older adult population is an urgent issue, especially in the context of avoiding co-epidemics during the current coronavirus disease 2019 crisis. The aim of this study was to examine factors associated with the quality of perceived patient–physician communication and whether this variable was associated with increased odds of vaccination.

**Methods:**

We used cross-sectional data from the Japan Gerontological Evaluation Study conducted from October 2016 to January 2017. The participants were 22,253 physically and cognitively independent individuals aged 65 or older living in 39 municipalities in Japan. Multilevel logit models were used to estimate the odds of vaccination.

**Results:**

Among the participants, 40.0% and 58.8% had received pneumococcal and influenza vaccinations as per the recommended schedule, respectively. People with low educational levels were more likely to have a family physician but rate their experience in asking questions lower than those with higher educational levels. Having a family physician and high rating for physicians’ listening attitude were positively associated with increased odds of pneumococcal and influenza vaccinations. High rating for patients’ questioning attitude and shared decision-making, compared to an ambiguous attitude toward medical decision-making, were positively associated with increased odds of pneumococcal vaccination.

**Conclusion:**

The results suggest that promotion of having a family physician, better patient–physician communication, and shared decision-making may encourage older adults to undergo recommended vaccinations.

## INTRODUCTION

Pneumococcal diseases and influenza pose a substantial health burden, especially among older adults.^1^^,^^2^ Vaccination for these diseases may reduce the risk of morbidity in this population.^3^^,^^4^ A routine single dose of a 23-valent polysaccharide vaccine and an annual influenza vaccination are, therefore, recommended for all persons aged 65 or older in the international guidelines developed by the World Health Organization and Centers for Disease Control and Prevention.^5^^,^^6^

In the present scenario of the coronavirus disease 2019 (COVID-19) pandemic, undergoing influenza vaccination is more crucial than ever for several reasons,^7^^,^^8^ and the same reasoning can apply to pneumococcal vaccination. First, COVID-19, pneumococcal diseases, and influenza are contagious respiratory illnesses that present with similar symptoms, making diagnosis difficult. Moreover, as older adults are vulnerable to these illnesses,^9^^,^^10^ medical and long-term care facilities have to be more careful of mass infections. Second, studies suggest that co-infection of COVID-19 with pneumococcal diseases or influenza may result in a more severe disease course, increased number of complications, and even death.^11^^–^^13^ Third, hospitalization for vaccine-preventable diseases could overwhelm the already limited medical resources available for patients with COVID-19. However, there is a gap between target rates and actual vaccine coverage for older adults in many developed countries.^14^ In Japan, the Immunization Act mandated municipalities to subsidize routine 23-valent polysaccharide vaccinations every 5 years and annual influenza vaccinations for persons aged 65 or older in 2014 and 2001, respectively. Nevertheless, vaccine coverage is suboptimal: 35.0% for pneumococcal vaccination and 48.2% for influenza vaccination.^15^

Physicians play an essential role in decision-making regarding preventive medical services. Systematic reviews point out that recommendation from physicians is a significant factor in the vaccination decision.^16^^,^^17^ Among adults aged 50–64 years, having a family physician (defined as a usual source of healthcare and advice) has been shown to be associated with higher odds of receiving preventive care, including influenza vaccination.^18^ The quality of patient–physician communication may also be influential. For example, high-quality patient–physician communication was positively associated with the receipt of meningococcal and human papillomavirus vaccination among adolescents aged 13–17,^19^ mammograms among women aged 40 or older,^20^ and colorectal cancer screening among persons aged 50 or older.^21^ However, few studies have examined the relationship between patient–physician communication and vaccination among older adults. Older patients need careful consultation because of the considerable heterogeneity in this population in terms of comorbidity, medical history, treatment options, and risks of adverse events.^22^^,^^23^ Nevertheless, some studies suggest that the quality of patient–physician communication in the context of older adults is not satisfactory.^24^^,^^25^ Hence, studying patient–physician communication with a focus on the older population is important to fill the knowledge gap.

In this study, we examined factors associated with the quality of perceived patient–physician communication and whether this variable was associated with increased odds of receiving pneumococcal and influenza vaccines using nationwide data of Japanese older adults aged 65 or older.

## METHODS

### Study participants

We analyzed cross-sectional data from the Japan Gerontological Evaluation Study (JAGES) in 2016. The JAGES is a large population-based study of Japanese people aged 65 or older who were physically and cognitively independent (ie, not needing public long-term care). Self-reported questionnaires were mailed to older adults in 39 municipalities from October 2016 to January 2017. Random sampling was employed in 22 large municipalities, while a census of all residents aged 65 or older was conducted in 17 smaller municipalities. Of 34,566 people invited to participate, 24,260 returned the questionnaires (response rate: 70.2%). Among the respondents, 2,002 were not eligible because they were certified as needing public long-term care. We also excluded five respondents who did not report their gender. Thus, our analytical sample comprised 22,253 individuals (10,180 men and 12,073 women; mean age, 74.2 years). All study participants provided informed consent, and the study protocol was reviewed and approved by ethics committees at the University of Tokyo (2019238NI), the National Center for Geriatrics and Gerontology (992), and Chiba University (2493).

### Outcome and explanatory variables

To identify whether a participant had undergone the recommended vaccinations, we asked, “Did you receive pneumococcal vaccination in the past 5 years?” and “Did you receive flu vaccination in the past year?”. If a participant answered “no,” they had to select all the following options that applied: “I will not get sick even without vaccination,” “I do not like injections;” “Vaccination is too expensive,” and “I did not know about the vaccination.”

The survey studied patient–physician communication in the following four domains. First, “having a family physician” corresponded to the question, “Do you have a family physician?”. In Japan, a family physician is not institutionalized like a general practitioner in the United Kingdom, and we defined it in the questionnaire as “the physician you go to when you have a health problem, and it does not matter what the physician’s specialty is.” Second, “physicians’ listening attitude” was evaluated by the question, “How would you rate how well your physician listened to you?”.^26^ We asked participants to recall their last visit to a physician and answer the question (the same applied to the following questions). Responses were recorded on a five-point Likert scale (1 = poor, 2 = fair, 3 = good, 4 = very good, 5 = excellent). Third, “patients’ questioning attitude” was assessed using the question, “How would you rate how well you were able to ask your physician about something you did not understand?”. Responses were recorded on a four-point Likert scale (1 = poor, 2 = fair, 3 = good, 4 = excellent). Fourth, “style of decision-making” was determined by the question, “How did you make a decision on your medical care?”. Based on the literature, we identified three communication styles^19^^,^^27^: (a) physician-driven decision making, called the “paternalistic” style, corresponding to the option “my physician decided everything”; (b) patient-driven decision making, called the “informed” style, corresponding to the option “I listened to my physician’s explanation and then made my own decision”; and (c) a mid-point on the continuum called the “shared” style, corresponding to the option “I listened to my physician’s explanation and then made a decision in consultation with him/her.” We also offered the option of “not sure.”

### Covariates

We adjusted for potential confounders, namely gender, age, education (low: ≤9 years, middle: 10–12 years, high: ≥13 years), marital status (married, other), engagement in paid work, annual equivalized household income (low: ≤1.9 million Japanese Yen [JPY], middle: 2–3.9 million JPY, high: ≥4 million JPY), receipt of public assistance based on the Public Assistance Act, self-reported diagnosis of diseases (heart disease, diabetes, respiratory disease, kidney or prostate gland disease, others), the copayment amount in the past month (did not visit a physician, 0 JPY, 1–4,999 JPY, 5,000–9,999 JPY, 10,000–19,999 JPY, 20,000 JPY or more), instrumental activities of daily living measured with the Tokyo Metropolitan Institute of Gerontology Index of Competence (fully capable: 5 points, less capable: ≤4 points),^28^ depressive symptoms assessed with the short form of the Geriatric Depression Scale (not depressed: ≤4 points, depressive tendency: 5–9 points, depression: ≥10 points),^29^ self-rated health measured with a four-point Likert scale (1 = poor, 2 = fair, 3 = good, 4 = excellent), smoking status, and perceived social capital at the individual level consisting of civic participation, social cohesion, and reciprocity.^30^ Civic participation was measured as the number of the following groups participants were involved in per month: volunteer groups, sports groups, hobby activities, study or cultural groups, and activities for teaching specific skills (none, one, two, over three). Social cohesion was determined using the number of participants who answered “strongly/moderately agree” on three questions about community trust, norms of reciprocity, and community attachment. Reciprocity was determined using the number of participants who answered “any one or more” on three questions about receiving and providing emotional support and receiving instrumental support. Total scores for each social capital variable ranged from 0 to 3.

### Statistical analysis

First, we explored socioeconomic and health-related factors associated with patient–physician communication using generalized linear models. A logit model was adopted for “having a family physician”; an ordinal logit model was adopted for “physicians’ listening attitude” and “patients’ questioning attitude”; and a multinomial logit model was adopted for “style of decision-making.” Second, we examined the associations between each domain of patient–physician communication and vaccination. To consider regional variations in vaccine coverage rates, we applied a multilevel logit model with random intercepts; individuals at level 1 were nested within municipalities at level 2. We show unadjusted and adjusted models with adjustment for the covariates shown above. Models for physicians’ listening attitude, patients’ questioning attitude, and style of decision making were also adjusted for based on whether one had a family physician. To address potential bias caused by missing values, we adopted multiple imputation under the missing at random assumption (ie, a missing mechanism is related to other variables measured in the same survey for that subject). Incomplete variables were imputed with a chained equation using all variables as explanatory variables. We created 10 datasets, and the estimates were combined. All analyses were performed using Stata version 16.1 (Stata Corp., College Station, TX, USA).

## RESULTS

Of the sample, 77.7% reported having a family physician (Table 1). The greatest number of participants rated their physicians’ listening attitude as “very good” (39.0%), followed by a large number of participants who rated it as “good” (28.7%). Most participants rated their experience in asking questions of their physician as “excellent” (31.8%) or “good” (45.5%). More than half of the participants (50.4%) made a decision on medical care in consultation with their physician, that is, in a “shared” style.

**Table 1.** Characteristics of participants (*N* = 22,253)

Variables	*n*	%	Mean (SD)
Men	10,180	45.8	
Age, years	22,253	100.0	74.2 (6.3)
Education			
Low	7,351	33.0	
Middle	8,922	40.1	
High	5,629	25.3	
Missing or others	351	1.6	
Marital status			
Married	15,689	70.5	
Others	6,235	28.0	
Missing	329	1.5	
Engagement in paid work			
Yes	5,294	23.8	
No	12,954	58.2	
Missing	4,005	18.0	
Household income			
Low	8,495	38.2	
Middle	6,728	30.2	
High	1,979	8.9	
Missing	5,051	22.7	
Receipt of public assistance^a^			
Yes	410	1.8	
No	21,528	96.7	
Missing	315	1.4	
Disease diagnosis			
Heart disease	2,148	9.7	
Diabetes	2,881	12.9	
Respiratory disease	1,152	5.2	
Kidney or prostate gland disease	1,548	7.0	
Others	10,675	48.0	
Missing	1,147	5.2	
Copayment			
No visit	1,932	8.7	
0 JPY	907	4.1	
1–4,999 JPY	9,431	42.4	
5,000–9,999 JPY	4,846	21.8	
10,000–19,999 JPY	1,916	8.6	
20,000 JPY or more	1,007	4.5	
Missing	2,214	9.9	
Instrumental activities of daily living			
Fully capable	19,351	87.0	
Less capable	2,192	9.9	
Missing	710	3.2	
Depressive symptoms			
Not depressed	14,115	63.4	
Depressive tendency	3,154	14.2	
Depression	938	4.2	
Missing	4,046	18.2	
Self-rated health	21,596	97.0	3.0 (0.6)
Smoking			
Yes	2,335	10.5	
No	19,391	87.1	
Missing	527	2.4	
Social capital			
Civic participation	16,401	73.7	0.8 (1.1)
Social cohesion	21,298	95.7	2.0 (1.1)
Reciprocity	21,080	94.7	2.8 (0.5)
Having a family physician			
Yes	17,280	77.7	
No	3,471	15.6	
Missing	1,502	6.7	
Physicians’ listening attitude			
Excellent	3,797	17.1	
Very good	8,676	39.0	
Good	6,391	28.7	
Fair	643	2.9	
Poor	165	0.7	
Missing	2,581	11.6	
Patients’ questioning attitude			
Excellent	7,081	31.8	
Good	10,118	45.5	
Fair	2,081	9.4	
Poor	161	0.7	
Missing	2,812	12.6	
Style of decision making			
Paternalistic	2,841	12.8	
Shared	11,224	50.4	
Informed	2,105	9.5	
Not sure	573	2.6	
Missing	5,510	24.8	

^a^“Receipt of public assistance” indicates those receiving public assistance based on the Public Assistance Act.

Coverage was 40.0% for pneumococcal vaccination and 58.8% for influenza vaccination (Table 2). Many participants who did not undergo flu vaccination stated, “I will not get sick even without it” (68.1%). In comparison, the proportions of participants who cited the same reason (37.4%) and who stated “I did not know about the vaccination” (30.6%) in the context of why they did not undergo pneumococcal vaccination were similar.

**Table 2.** Vaccination and reasons of not receiving it

Variables	*n*	%
*Pneumococcal vaccination*		
Yes	8,893	40.0
No	11,516	51.8
Missing	1,844	8.3
Reasons of not receiving the vaccination (*n* = 11,516):		
I will not get sick even without it.	4,309	37.4
I do not like injections.	899	7.8
It is too expensive.	567	4.9
I did not know the vaccination.	3,524	30.6

*Influenza vaccination*		
Yes	13,015	58.5
No	8,023	36.1
Missing	1,215	5.5
Reasons of not receiving the vaccination (*n* = 8,023):		
I will not get sick even without it.	5,461	68.1
I do not like injections.	1,194	14.9
It is too expensive.	485	6.0
I did not know the vaccination.	497	6.2

Table 3 presents socioeconomic and health-related factors associated with patient–physician communication. Age was positively associated with the odds of having a family physician, higher ratings for physicians’ listening attitude and patients’ questioning attitude. Further, older participants were less likely to experience shared and informed decision-making. People with low educational levels were more likely to have a family physician and rate their experience of asking questions lower compared to those with high education.

**Table 3.** Generalized linear models for socioeconomic and health-related determinants of patient–physician communication^a^ (*N* = 22,253)

Variables	Having a family physician			Physicians’ listening attitude			Patients’ questioning attitude			Style of decision making								

										Paternalistic			Shared			Informed		
	OR	95% CI		OR	95% CI		OR	95% CI		RR	95% CI		RR	95% CI		RR	95% CI	
Having a family physician	—			2.46	2.26	2.67	2.44	2.24	2.67	1.83	1.43	2.34	2.12	1.70	2.65	1.29	1.001	1.65
Men	0.65	0.59	0.71	0.95	0.89	1.02	0.97	0.91	1.04	1.81	1.46	2.24	1.50	1.22	1.85	1.33	1.06	1.66
Age	1.07	1.06	1.08	1.03	1.02	1.03	1.02	1.02	1.03	1.01	0.99	1.02	0.97	0.95	0.98	0.97	0.95	0.99
Education^b^																		
Low	1.47	1.31	1.65	1.03	0.95	1.11	0.79	0.73	0.85	0.68	0.52	0.89	0.55	0.43	0.70	0.52	0.40	0.68
Middle	1.13	1.02	1.25	0.97	0.91	1.04	0.89	0.82	0.95	0.90	0.70	1.15	0.78	0.61	0.99	0.77	0.59	0.99
Married	1.11	1.01	1.23	0.88	0.82	0.95	0.88	0.82	0.94	0.91	0.72	1.16	0.96	0.78	1.18	0.92	0.72	1.17
Paid work	1.14	1.03	1.25	1.07	0.996	1.15	1.03	0.95	1.11	0.94	0.72	1.23	0.85	0.65	1.12	0.92	0.72	1.18
Household income^b^																		
Low	0.97	0.84	1.11	1.02	0.92	1.14	0.89	0.81	0.98	1.22	0.84	1.79	0.89	0.62	1.26	1.01	0.66	1.54
Middle	0.98	0.85	1.12	0.97	0.88	1.07	0.93	0.84	1.02	1.21	0.86	1.69	1.04	0.75	1.45	1.05	0.71	1.53
Receipt of public assistance^c^	1.81	1.31	2.49	1.57	1.24	1.98	1.41	1.13	1.77	1.35	0.70	2.60	0.90	0.49	1.62	0.71	0.38	1.32
Disease diagnosis																		
Heart disease	4.40	3.60	5.40	1.13	1.03	1.24	1.08	0.97	1.21	1.42	0.91	2.20	1.46	0.94	2.25	1.49	0.96	2.34
Diabetes	4.19	3.47	5.06	1.12	1.02	1.22	1.04	0.93	1.15	1.45	0.97	2.17	1.33	0.93	1.90	1.20	0.78	1.85
Respiratory disease	3.23	2.48	4.20	1.05	0.93	1.20	1.01	0.88	1.17	2.09	1.19	3.65	2.19	1.22	3.94	1.96	1.09	3.53
Kidney disease	2.36	1.93	2.88	0.99	0.88	1.11	0.96	0.85	1.09	2.07	1.21	3.54	2.55	1.50	4.35	1.99	1.12	3.56
Others	3.16	2.82	3.53	0.96	0.89	1.04	0.94	0.85	1.03	0.91	0.68	1.22	0.99	0.78	1.25	0.96	0.75	1.24
Copayment^b^																		
0 JPY	1.82	1.49	2.22	1.41	1.19	1.67	1.49	1.25	1.77	1.70	1.13	2.56	1.42	0.97	2.07	1.37	0.89	2.13
1–4,999 JPY	3.97	3.52	4.49	1.08	0.97	1.21	1.19	1.06	1.32	2.20	1.64	2.96	2.25	1.70	2.97	2.03	1.52	2.70
5,000–9,999 JPY	4.60	3.95	5.36	1.17	1.03	1.32	1.23	1.08	1.39	2.07	1.47	2.93	2.12	1.56	2.89	1.80	1.29	2.49
10,000–19,999 JPY	4.93	3.99	6.10	1.36	1.18	1.57	1.34	1.16	1.54	1.90	1.24	2.91	1.89	1.29	2.79	1.80	1.18	2.73
20,000 JPY or more	4.54	3.58	5.76	1.58	1.35	1.84	1.43	1.21	1.70	2.46	1.46	4.15	2.24	1.38	3.63	2.17	1.26	3.73
Capable of IADL^c^	0.85	0.73	0.98	1.00	0.91	1.11	1.29	1.17	1.42	0.94	0.69	1.28	1.34	1.001	1.80	1.41	1.03	1.93
Depressive symptoms^b^																		
Depressive tendency	0.82	0.72	0.92	0.75	0.69	0.82	0.64	0.58	0.70	0.75	0.57	0.99	0.63	0.48	0.83	0.75	0.56	1.00
Depression	0.65	0.53	0.80	0.61	0.52	0.71	0.48	0.41	0.57	0.65	0.44	0.95	0.46	0.32	0.65	0.57	0.38	0.87
Self-rated health^c^	1.13	1.04	1.22	1.27	1.21	1.34	1.34	1.26	1.42	1.06	0.90	1.26	0.98	0.84	1.15	1.07	0.90	1.26
Smoking^c^	0.81	0.71	0.91	1.14	1.04	1.26	1.32	1.19	1.47	1.06	0.76	1.48	1.02	0.76	1.37	1.06	0.76	1.48
Social capital^c^																		
Civic participation	1.04	0.99	1.08	0.92	0.90	0.95	0.94	0.91	0.98	0.99	0.88	1.10	1.16	1.04	1.29	1.18	1.05	1.32
Social cohesion	1.16	1.12	1.21	1.21	1.18	1.25	1.14	1.11	1.17	1.20	1.09	1.32	1.26	1.15	1.38	1.15	1.05	1.26
Reciprocity	1.20	1.11	1.30	1.15	1.08	1.22	1.20	1.13	1.27	1.42	1.26	1.61	1.73	1.53	1.96	1.60	1.40	1.84

CI, confidence interval; IADL, instrumental activities of daily living; JPY, Japanese Yen; OR, odds ratio; RR, risk ratio.

^a^A logit model is used for having a family physician, an ordinal logit model is used for physicians’ listening attitude and patients’ questioning attitude, and a multinomial logit model is used for style of decision making. The reference category for the multinomial logit model is “not sure.”

^b^The reference category is “high” for education and household income, “no visit” for copayment, and “not depressed” for depressive symptoms.

^c^“Receipt of public assistance” indicates those receiving public assistance based on the Public Assistance Act; “Capable of IADL” indicates those who are fully capable of IADL; “Self-rated health” is measured with a four-point Likert scale; “Smoking” indicates those who are currently smoking; three sub-measures of “Social capital” represent scores ranged from 0 to 3.

Table 4 presents the associations between patient–physician communication and vaccination (for the other covariates, see eTable 1). We separately conducted regressions for each of the four domains in patient–physician communication. In the unadjusted models, only the variable of having a family physician was clearly associated with increased odds of pneumococcal vaccination. After adjusting for possible confounders, having a family physician (odds ratio [OR] 1.66; 95% confidence interval [CI], 1.51–1.82), physicians’ listening attitude (OR 1.44; 95% CI, 1.02–2.03; excellent vs poor), patients’ questioning attitude (OR 1.48; 95% CI, 1.04–2.12; excellent vs poor), and the shared decision-making style (OR 1.22; 95% CI, 1.01–1.47; compared to an ambiguous attitude) were associated with increased odds of pneumococcal vaccination. On the other hand, influenza vaccination was positively associated with all four domains in patient–physician communication in the unadjusted models. After adjusting for confounders, the associations attenuated, though the directions of point estimates were similar; having a family physician (OR 2.28; 95% CI, 2.09–2.49) and physicians’ listening attitude (OR 1.59; 95% CI, 1.13–2.25; excellent vs poor) were associated with increased odds of influenza vaccination. In addition, compared to an ambiguous attitude toward medical decision-making, the informed decision-making style was associated with decreased odds of influenza vaccination (OR 0.82; 95% CI, 0.67–0.999). To examine whether the status of having a family physician modifies the associations, we checked the interaction terms between having a family physician and the other patient–physician communication variables (ie, physicians’ listening attitude, patients’ questioning attitude, and style of decision making). These interaction terms did not appear to be associated with vaccination (see eTable 2), which suggests that the associations between patient–physician communication and vaccination were not different between the group that had a family physician and the group that did not.

**Table 4.** Multilevel logit models for associations between patient–physician communication and vaccination (*N* = 22,253)

	Pneumococcal vaccination						Influenza vaccination					
	Unadjusted			Adjusted^a^			Unadjusted			Adjusted^a^		
	OR	95% CI		OR	95% CI		OR	95% CI		OR	95% CI	
*Having a family physician*												
Yes	2.22	2.05	2.41	1.66	1.51	1.82	3.39	3.15	3.65	2.28	2.09	2.49
No	Ref.			Ref.			Ref.			Ref.		

*Physicians’ listening attitude*												
Excellent	0.95	0.87	1.05	1.44	1.02	2.03	2.22	1.98	2.47	1.59	1.13	2.25
Very good	0.86	0.79	0.93	1.41	1.004	1.97	1.91	1.73	2.10	1.56	1.11	2.18
Good	0.80	0.73	0.87	1.47	1.05	2.05	1.55	1.41	1.72	1.51	1.08	2.11
Fair	0.68	0.57	0.80	1.40	0.97	2.02	1.27	1.06	1.53	1.44	0.99	2.08
Poor	Ref.			Ref.			Ref.			Ref.		

*Patients’ questioning attitude*												
Excellent	0.92	0.85	1.00	1.48	1.04	2.12	2.03	1.84	2.25	1.10	0.77	1.58
Good	0.84	0.78	0.91	1.48	1.03	2.12	1.68	1.52	1.85	1.04	0.73	1.49
Fair	0.68	0.61	0.76	1.37	0.95	2.00	1.28	1.13	1.44	0.93	0.65	1.33
Poor	Ref.			Ref.			Ref.			Ref.		

*Style of decision making*												
Paternalistic	0.86	0.76	0.97	1.03	0.84	1.26	1.60	1.43	1.78	0.98	0.81	1.18
Shared	1.03	0.93	1.13	1.22	1.01	1.47	1.73	1.58	1.89	1.08	0.90	1.29
Informed	0.75	0.66	0.87	0.97	0.78	1.19	1.20	1.06	1.35	0.82	0.67	0.999
Not sure	Ref.			Ref.			Ref.			Ref.		

CI confidence interval; OR, odds ratio.

^a^Adjusted for gender, age, education, marital status, paid work, household income, receipt of public assistance, self-reported diagnosis of diseases, the amount of copayment, instrumental activities of daily living, depressive symptoms, self-rated health, smoking status, and social capital. Models for physicians’ listening attitude, patients’ questioning attitude, and style of decision making are also adjusted for based on whether one has a family physician.

## DISCUSSION

This study explored the association between patient–physician communication and vaccination in Japanese older adults. The results show that having a family physician, positive perceptions of physicians’ listening attitude and their own questioning ability, and shared decision-making were associated with increased odds of pneumococcal vaccination among people aged 65 or older. We found similar associations with influenza vaccination.

In a previous study, a conceptual model was used to describe how positive patient–physician communication can lead to receipt of preventive medical services.^31^ Patients may want their physician to listen to their descriptions of their symptoms or explain what they do not understand. Exchanging information in a way the patient understands promotes patient knowledge and understanding and leads to greater trust in the physician. It also fosters self-efficacy and feelings of empowerment, which lead to improved treatment adherence, health habits, and self-care. Thus, good patient–physician relationships encourage patients to follow through with recommended treatment, and in turn, induce timely receipt of preventive care. While this pathway has been observed in previous studies, they mostly pertained to younger patients.^19^^,^^21^^,^^32^ Although a previous study did not find a clear association between patient–physician communication and influenza vaccination for persons aged 50 or older,^20^ the present study suggests that the theory can, in fact, apply to vaccination in the older population (Table 4). Family physicians play an important role in the uptake of the recommended vaccination in the older population because they can build close relationships with their patients, and because older patients are more likely to have a family physician than younger patients.^33^ In line with another report,^33^ 77.7% of our participants had a family physician (Table 1). In our participants, overconfidence that they could not be infected was the most common reason for not undergoing vaccination (Table 2). Appropriate communication would eliminate any such misconceptions and help patients understand the need for vaccination. Provision of information from a physician is also essential to increase the uptake of pneumococcal vaccination because many people are not aware of it.

We tested various decision-making styles and found that shared decision-making was most likely to be associated with increased odds of pneumococcal vaccination (Table 4). In previous studies, however, the paternalistic decision-making style was associated with increased odds of meningococcal and human papillomavirus vaccination among adolescents aged 13–17^19^ and of parents’ acceptance of vaccinations for their children^34^^,^^35^ rather than shared or informed decision-making. We found a negative association between informed decision-making and influenza vaccination, which may be in line with these previous findings. In contrast, the patients’ age may explain the positive association between shared decision-making and pneumococcal vaccination in this study. Prior research has reported that preference for shared decision-making increases with age.^36^ Shared decision-making promotes information exchange in a way that addresses patients’ needs,^27^ and being treated in the preferred way fosters trust in a physician and may increase vaccination acceptance. Involving older patients in the decision-making process may increase vaccination uptake especially for pneumococcal vaccination, which is not well known.

The present study also explored socioeconomic and health-related factors associated with patient–physician communication (Table 3). In accordance with previous studies, we found that age was positively associated with having a family physician and higher ratings for physicians’ listening attitude and patients’ questioning attitude.^37^^–^^39^ This may be because older adults tend to visit physicians more frequently than other age groups, giving them more opportunities to build close relationships.^37^ The same logic can apply to more favorable perceptions among participants who had some diseases and who paid more copayments. Evidence regarding the association between educational attainment and quality of perceived patient–physician communication has been mixed.^37^^–^^39^ Our results showed that people with higher education tended to rate their experience in asking questions higher but physicians’ listening attitude lower compared to those with low educational levels. This may be interpreted as indicating that education and increased knowledge enabled patients to ask questions of their physicians, but at the same time, their raised expectations of their physicians were not met.^37^ We also found that people with low educational levels were more likely to have a family physician. This suggests that family physicians play an essential role in helping them understand the need for vaccination and in reducing social disparity in vaccination rates across different levels of education.

This study has several limitations. First, we asked participants to recall their communication during their last visit to a physician. Hence, we could not evaluate the quality of communication when a participant decided to undergo vaccination. In addition, healthy participants may experience difficulty recalling their last communication with a physician because they seldom visit a physician and talk about their health problems. Further, we did not seek to understand physicians’ perspective, despite the fact that their confidence in and willingness to recommend vaccines affect patient acceptance.^16^ Further studies with close observations of communication on vaccination between physician and older patients are needed. Second, although our questions on patient–physician communication were similar to those used in studies such as the Community Tracking Study^26^ and Health Information National Trends Survey, the Japanese questionnaire has not been validated. A previous study reported that only 14.6% Japanese physicians adopted shared decision making, whereas 58% of American physicians decision making for lifestyle modification.^40^ More than half of our participants reported that they experienced shared decision making, which is similar to the American sample rather than the Japanese sample. There are possibilities of measurement errors and misclassification. Third, the culture of patient–physician communication, attitudes toward vaccination, and healthcare systems vary across countries. The generalizability of our findings, therefore, may be limited. Fourth, we could not obtain some important covariates, such as characteristics of the institutions the participants visited, disease severities, and the frequency of physician visits. Although we adjusted for self-reported disease diagnoses, the amount of copayment in the past month, instrumental activities of daily living, and self-rated health, which can partially surrogate the omitted variables, bias could remain if the adjustment was not sufficient.

In summary, this study found that pneumococcal vaccination for older adults aged 65 and above was positively associated with having a family physician, physicians’ listening attitude, patients’ questioning attitude, and shared decision-making. Influenza vaccination was also positively associated with having a family physician and the physicians’ listening attitude. The results suggest that promotion of having a family physician, better patient–physician communication, and shared decision-making may encourage older adults to undergo recommended vaccinations.

## SUPPLEMENTARY MATERIAL

eTable 1. Multilevel logit models for associations between patient-physician communication and vaccination

eTable 2. Multilevel logit models for the associations of vaccination with interaction terms between patient-physician communication and having a family physician^a^
