# Supplementary material for: Association of Pneumococcal and Influenza Vaccination With Patient–Physician Communication in Older Adults: A Nationwide Cross-sectional Study From the JAGES 2016
Source: J Epidemiol. 2022 Sep 5;32(9):401–7. doi: 10.2188/jea.JE20200505 (PMC9359899; doi:10.2188/jea.JE20200505)
Supplement: Supplementary file 1 [file je-32-401-s001.pdf]

eTable 1. Multilevel logit models for associations between patient-physician communication and vaccination

Variables	Pneumococcal vaccination											
	Having a family physician			*Physicians' listening attitude*			*Patients' questioning attitude*			*Style of decision making*		
	OR	95% CI		OR	95% CI		OR	95% CI		OR	95% CI	
Having a family physician												
Yes	1.66	1.51	1.82	1.66	1.51	1.82	1.64	1.49	1.80	1.63	1.48	1.79
No	Ref.											
Physicians' listening attitude												
Excellent				1.44	1.02	2.03						
Very good				1.41	1.004	1.97						
Good				1.47	1.05	2.05						
Fair				1.40	0.97	2.02						
Poor				Ref.								
Patients' questioning attitude												
Excellent							1.48	1.04	2.12			
Good							1.48	1.03	2.12			
Fair							1.37	0.95	2.00			
Poor							Ref.					
Style of decision making												
Paternalistic										1.03	0.84	1.26
Shared										1.22	1.01	1.47
Informed										0.97	0.78	1.19
Not sure										Ref.		
Men	0.88	0.83	0.94	0.88	0.83	0.94	0.88	0.83	0.94	0.88	0.83	0.94
Age	1.04	1.03	1.04	1.04	1.03	1.04	1.04	1.03	1.04	1.04	1.03	1.04
Education [a]												
Low	0.99	0.90	1.08	0.99	0.90	1.08	0.99	0.91	1.09	0.99	0.91	1.09
Middle	1.04	0.96	1.12	1.04	0.96	1.12	1.04	0.96	1.12	1.04	0.96	1.12
Married	1.03	0.96	1.11	1.03	0.96	1.11	1.03	0.96	1.11	1.03	0.96	1.11
Paid work	0.92	0.86	1.00	0.92	0.86	1.00	0.92	0.86	1.00	0.93	0.86	0.9996
Household income [a]												
Low	0.88	0.77	0.99	0.87	0.77	0.99	0.88	0.78	0.99	0.88	0.78	0.997
Middle	0.98	0.88	1.10	0.98	0.88	1.10	0.98	0.88	1.10	0.99	0.88	1.10
Receipt of public assistance	0.95	0.74	1.22	0.95	0.74	1.22	0.95	0.74	1.22	0.95	0.74	1.23
Disease diagnosis												

Heart disease	1.14	1.03	1.27	1.14	1.03	1.27	1.14	1.03	1.27	1.14	1.03	1.27
Diabetes	1.06	0.96	1.17	1.06	0.96	1.17	1.06	0.96	1.17	1.06	0.96	1.17
Respiratory disease	1.95	1.70	2.24	1.95	1.70	2.24	1.95	1.70	2.24	1.94	1.69	2.23
Kidney disease	1.25	1.10	1.42	1.25	1.10	1.42	1.25	1.10	1.42	1.24	1.09	1.41
Others	1.09	1.01	1.18	1.09	1.01	1.18	1.09	1.01	1.19	1.09	1.01	1.18
Copayment [a]												
0 JPY	1.44	1.18	1.76	1.44	1.19	1.76	1.44	1.18	1.75	1.44	1.19	1.75
1–4,999 JPY	1.61	1.41	1.84	1.61	1.41	1.84	1.61	1.41	1.84	1.60	1.40	1.83
5,000–9,999 JPY	2.00	1.74	2.31	2.00	1.74	2.30	2.00	1.74	2.30	1.99	1.73	2.29
10,000–19,999 JPY	2.10	1.78	2.47	2.10	1.78	2.47	2.09	1.77	2.46	2.09	1.77	2.46
20,000 JPY or more	1.92	1.60	2.30	1.93	1.61	2.31	1.92	1.60	2.30	1.92	1.60	2.30
Capable of IADL	1.13	1.02	1.25	1.13	1.02	1.25	1.12	1.01	1.24	1.12	1.01	1.24
Depressive symptoms [a]												
Depressive tendency	0.89	0.82	0.98	0.90	0.82	0.98	0.90	0.83	0.98	0.90	0.83	0.98
Depression	0.85	0.72	1.00	0.85	0.72	1.01	0.86	0.73	1.02	0.86	0.73	1.02
Self-rated health	1.01	0.96	1.07	1.01	0.96	1.08	1.01	0.95	1.07	1.02	0.96	1.08
Smoking	0.69	0.62	0.77	0.69	0.62	0.77	0.69	0.62	0.77	0.69	0.62	0.77
Social capital												
Civic participation	1.07	1.03	1.10	1.07	1.03	1.10	1.07	1.03	1.10	1.06	1.03	1.10
Social cohesion	1.02	0.99	1.05	1.02	0.99	1.05	1.02	0.99	1.05	1.02	0.99	1.05
Reciprocity	1.16	1.09	1.25	1.16	1.08	1.25	1.16	1.08	1.24	1.15	1.08	1.24

Variables	Influenza vaccination											
	Having a family physician			*Physicians' listening attitude*			*Patients' questioning attitude*			*Style of decision making*		
	OR	95% CI		OR	95% CI		OR	95% CI		OR	95% CI	
Having a family physician												
Yes	2.28	2.09	2.49	2.24	2.05	2.45	2.23	2.04	2.43	2.24	2.06	2.45
No	Ref.											
Physicians' listening attitude												
Excellent				1.59	1.13	2.25						
Very good				1.56	1.11	2.18						
Good				1.51	1.08	2.11						
Fair				1.44	0.99	2.08						
Poor				Ref.								
Patients' questioning attitude												
Excellent							1.10	0.77	1.58			

Good							1.04	0.73	1.49			
Fair							0.93	0.65	1.33			
Poor							Ref.					
Style of decision making												
Paternalistic										0.98	0.81	1.18
Shared										1.08	0.90	1.29
Informed										0.82	0.67	0.999
Not sure										Ref.		
Men	0.69	0.64	0.74	0.69	0.65	0.74	0.69	0.64	0.74	0.69	0.64	0.74
Age	1.06	1.06	1.07	1.06	1.06	1.07	1.06	1.06	1.07	1.07	1.06	1.07
Education [a]												
Low	1.15	1.05	1.25	1.14	1.05	1.25	1.15	1.05	1.26	1.15	1.05	1.25
Middle	1.07	0.99	1.15	1.07	0.98	1.15	1.07	0.99	1.16	1.07	0.99	1.15
Married	1.02	0.95	1.10	1.02	0.95	1.10	1.02	0.95	1.10	1.02	0.95	1.10
Paid work	1.11	1.02	1.20	1.10	1.02	1.19	1.11	1.03	1.19	1.11	1.03	1.20
Household income [a]												
Low	0.98	0.87	1.09	0.98	0.87	1.09	0.98	0.88	1.10	0.98	0.88	1.10
Middle	1.03	0.93	1.14	1.03	0.93	1.14	1.03	0.93	1.15	1.03	0.93	1.15
Receipt of public assistance	1.08	0.85	1.36	1.07	0.85	1.36	1.07	0.84	1.35	1.08	0.85	1.36
Disease diagnosis												
Heart disease	1.25	1.11	1.40	1.24	1.11	1.39	1.24	1.11	1.39	1.25	1.11	1.40
Diabetes	1.19	1.06	1.32	1.18	1.06	1.32	1.19	1.06	1.32	1.18	1.06	1.32
Respiratory disease	1.56	1.33	1.83	1.56	1.33	1.83	1.56	1.33	1.83	1.56	1.32	1.83
Kidney disease	1.36	1.18	1.55	1.36	1.18	1.55	1.36	1.19	1.55	1.35	1.18	1.54
Others	1.17	1.07	1.28	1.17	1.07	1.28	1.17	1.07	1.28	1.17	1.07	1.28
Copayment [a]												
0 JPY	1.49	1.24	1.79	1.48	1.24	1.78	1.48	1.23	1.77	1.49	1.24	1.79
1–4,999 JPY	1.85	1.64	2.09	1.85	1.63	2.09	1.84	1.63	2.08	1.84	1.63	2.09
5,000–9,999 JPY	2.34	2.06	2.67	2.34	2.05	2.67	2.34	2.05	2.66	2.33	2.04	2.66
10,000–19,999 JPY	2.55	2.16	3.02	2.54	2.15	3.01	2.54	2.15	3.00	2.55	2.15	3.02
20,000 JPY or more	2.12	1.76	2.54	2.11	1.75	2.53	2.10	1.75	2.53	2.12	1.76	2.54
Capable of IADL	1.05	0.94	1.16	1.05	0.94	1.16	1.04	0.94	1.16	1.04	0.94	1.16
Depressive symptoms [a]												
Depressive tendency	0.92	0.84	1.01	0.93	0.85	1.02	0.93	0.85	1.02	0.93	0.85	1.02
Depression	0.96	0.81	1.13	0.97	0.82	1.14	0.98	0.83	1.16	0.97	0.82	1.15

Self-rated health	0.98	0.92	1.03	0.97	0.92	1.03	0.97	0.91	1.03	0.98	0.92	1.04
Smoking	0.70	0.63	0.77	0.70	0.63	0.77	0.70	0.63	0.77	0.70	0.63	0.77
Social capital												
Civic participation	1.10	1.06	1.14	1.10	1.06	1.14	1.10	1.06	1.14	1.10	1.06	1.14
Social cohesion	1.03	1.00	1.06	1.02	0.99	1.06	1.02	0.99	1.06	1.02	0.99	1.06
Reciprocity	1.20	1.12	1.28	1.19	1.12	1.27	1.19	1.12	1.28	1.19	1.12	1.28

CI, confidence interval; IADL, instrumental activities of daily living; JPY, Japanese Yen; OR, odds ratio; RR, risk ratio.

[a] The reference category is "high" for education and household income, "no visit" for copayment, and "not depressed" for depressive symptoms.

eTable 2. Multilevel logit models for the associations of vaccination with interaction terms between patient-physician communication and having a family physician [a]

	Pneumococcal vaccination			Influenza vaccination		
	OR	95% CI		OR	95% CI	
Physicians' listening attitude						
Excellent	0.98	0.51	1.87	1.13	0.62	2.07
Very good	1.31	0.71	2.41	1.34	0.76	2.34
Good	1.46	0.80	2.66	1.44	0.84	2.47
Fair	1.46	0.74	2.87	1.37	0.76	2.47
Having a family physician	1.52	0.74	3.10	1.89	0.96	3.73
Excellent * Family physician	1.55	0.72	3.31	1.53	0.74	3.14
Very good * Family physician	1.11	0.54	2.31	1.25	0.62	2.51
Good * Family physician	1.03	0.50	2.12	1.09	0.55	2.17
Fair * Family physician	0.94	0.41	2.14	1.08	0.51	2.28
Patients' questioning attitude						
Excellent	1.06	0.55	2.04	0.82	0.45	1.50
Good	1.30	0.67	2.51	0.95	0.53	1.69
Fair	1.18	0.60	2.34	0.92	0.52	1.64
Having a family physician	1.24	0.56	2.75	1.82	0.88	3.74
Excellent * Family physician	1.57	0.70	3.51	1.49	0.70	3.17
Good * Family physician	1.25	0.56	2.79	1.19	0.57	2.48
Fair * Family physician	1.27	0.56	2.88	1.05	0.50	2.22
Style of decision making						
Paternalistic	1.08	0.70	1.68	1.12	0.76	1.65
Shared	1.41	0.93	2.13	1.27	0.88	1.82
Informed	1.06	0.68	1.65	0.96	0.65	1.42
Having a family physician	1.90	1.19	3.04	2.79	1.85	4.22
Paternalistic * Family physician	0.92	0.57	1.49	0.82	0.50	1.33
Shared * Family physician	0.83	0.51	1.34	0.79	0.51	1.22
Informed * Family physician	0.88	0.53	1.47	0.79	0.49	1.25

CI confidence interval; OR, odds ratio.

[a] All models are adjusted for gender, age, education, marital status, paid work, household income, receipt of public assistance, self-reported diagnosis of diseases, the amount of copayment, instrumental activities of daily living, depressive symptoms, self-rated health, smoking status, and social capital.
